# Chromosomal variants accumulate in genomes of the spontaneous aborted fetuses revealed by chromosomal microarray analysis

**DOI:** 10.1371/journal.pone.0259518

**Published:** 2021-11-02

**Authors:** Sen Li, Lei-Ling Chen, Xing-Hua Wang, Hai-Jing Zhu, Xiao-Long Li, Xie Feng, Lei Guo, Xiang-Hong Ou, Jun-Yu Ma

**Affiliations:** 1 Guangdong-Hong Kong Metabolism & Reproduction Joint Laboratory, Guangdong Second Provincial General Hospital, Guangzhou, China; 2 Reproductive Medicine Center, Guangdong Second Provincial General Hospital, Guangzhou, China; 3 Bioland Laboratory, Guangzhou Regenerative Medicine and Health Guangdong Laboratory, Guangzhou, China; Nanjing Agricultural University, CHINA

## Abstract

Spontaneous abortion is an impeding factor for the success rates of human assistant reproductive technology (ART). Causes of spontaneous abortion include not only the pregnant mothers’ health conditions and lifestyle habits, but also the fetal development potential. Evidences had shown that fetal chromosome aneuploidy is associated with fetal spontaneous abortion, however, it is still not definite that whether other genome variants, like copy number variations (CNVs) or loss of heterozygosity (LOHs) is associated with the spontaneous abortion. To assess the relationship between the fetal genome variants and abortion during ART, a chromosomal microarray data including chromosomal information of 184 spontaneous aborted fetuses, 147 adult female patients and 78 adult male patients during ART were collected. We firstly analyzed the relationship of fetal aneuploidy with maternal ages and then compared the numbers and lengths of CNVs (< 4Mbp) and LOHs among adults and aborted fetuses. In addition to the already known association between chromosomal aneuploidy and maternal ages, from the chromosomal microarray data we found that the numbers and the accumulated lengths of short CNVs and LOHs in the aborted fetuses were significantly larger or longer than those in adults. Our findings indicated that the increased numbers and accumulated lengths of CNVs or LOHs might be associated with the spontaneous abortion during ART.

## Introduction

Epidemiological data had revealed that up to 10% clinically recognized pregnancies will spontaneously aborted [1–3]. For women who were undergoing assistant reproduction, an even bigger abortion risk (14.7%) would occur [4]. As 8–12% couples worldwide will be affected by infertility [5], and the assistant reproductive technology (ART) cycles had reached 2.0 million each year [6], so the ART caused abortion will affect more and more couples. So investigations about the causing factors of spontaneous abortion, especially ART associated abortion, is of important for modern obstetrics and for improving the success rates of ART. Previous evidences had shown that about half of spontaneous abortion were caused by the chromosome abnormalities [7, 8], including aneuploidy and/or polyploidy such as trisomy, monosomy and tetraploidy. Generally, the aneuploidy in fetuses could be originated from oocyte, whose aneuploid rate can range from 10–20% to more than 60% with the increase of maternal age [9]. Aneuploidy could also generated in mitotic blastomeres which account for more than 25% of the aneuploid embryos [9] or more than half of the mosaic aneuploid embryos [10]. These abortion induced by chromosome numerical abnormality might be associated with the dysregulation of dose-dependent genes during fetus development.

In addition to the numerical abnormality of chromosomes, new fetus could also inherit copy number variations (CNVs) or obtain loss of heterozygosity (LOH) from the parental genomes, and these CNVs and LOHs might be detrimental to the embryonic or fetal development. CNVs, including CNV gains and losses, are the common types of genome rearrangements. Generally, CNVs could be divided into recurrent CNVs and rare CNVs. The recurrent CNVs are caused by non-allele homologous recombination repair of DNA DSBs at the low copy repeat (LCR) regions [11, 12]. When DSB was produced at the LCR region, the overhang of the resected DSB end might invade the non-allele repeat with the same direction and after crossover repair the DNA sequence between the direct repeats might be removed or duplicated [11]. In other cases, when DBSs formed at the non-LCR regions when DNA replication encounter stress or after irradiation, the non-homologous recombination repair (such as non-homologous end joining [13] or break-induced replication [14]) of these DSBs could foster the formation of rare CNV whose frequency in human population were less than 0.1% [15]. The fetal CNVs inherited from parents could be either innate in their parental genomes or *de novo* formed in their parents’ gametes. Unlike CNVs, the LOHs in fetus can be induced by consanguineous marriages or marriages between relative close relatives. In addition, during the homologous recombination repair of DNA DSBs, broken DNA ends might use the non-sister homologous chromatid as template to repair the DSBs, which might induce large sequence of broken DNA replaced by the homologous sequence (termed as gene conversion) [16, 17]. The transitions of homologous DNA sequence then induce the LOHs in the genome [18] which could be detected by the chromosomal microarray based on runs of SNPs where heterozygote calls are absent. So the germline LOHs might be either produced by marriages of close related parents or *de novo* generated by DSB abnormal repair during early embryo development. Accumulated data revealed that both CNVs and LOHs could be pathogenic, benign or minor-effective for individuals [19, 20]. However, the increased number and accumulated length of these genome abnormalities might increase pathogenic risks based on the multiple-hit model [21].

To investigate whether the CNV or LOH burden are associated with spontaneous abortion in euploid fetus and the differences of CNVs and LOHs between the ART-aborted fetuses and adults, we analyzed the chromosomal microarray data of aborted fetuses during ART, and the chromosomal microarray data of ART patients were used as controls. Our results revealed that the numbers and accumulated lengths of CNVs and LOHs were increased in the aborted euploid fetuses.

## Materials and methods

### Ethical statement

Ethics approval for the data collection in this project was obtained from the Medical Ethics Committee of Guangdong Second Provincial General Hospital. The chromosomal microarray analysis (CMA) data were originated from the chorion samples of the aborted fetuses of the ART mothers (ART-aborted fetuses) and blood samples of adults undergone ART in the Guangdong Second Provincial General Hospital from 2017.05 to 2018.11, and the study was conducted on 2021.08.13. The information that could identify individual participants hadn’t been accessed by authors during or after data collection and the ethics committee waived the requirement of informed consent for this retrospective study.

### Data collection and analysis

Genomic DNA were isolated from the blood samples of ART patients and chorion samples of aborted fetuses using the QIAamp DNA Mini kit (Qiagen, Valencia, USA). Then these genomic DNA were fragmented according the instructions of Affymetrix GeneChip platform (Thermo Fisher Scientific, USA) and the DNA fragment products were hybridized with the CytoScan 750K Array chips. Then the chips were washed and scanned by GeneChip Scanner 3000 7G (Thermo Fisher Scientific, USA). After that, the raw data generated by the scanner were analyzed using the Chromosome Analysis Suite (ChAS) v4.0 software. UCSC hg19 were used as the reference genome in this study [22]. The chromosomal gains, losses and LOHs were detected by the ChAS software under the default parameters. The CNV data from dbVar were downloaded from dbVar databases [23]. All the data about the lengths or the counts of CNVs and LOHs in this study were calculated using the R software. All data visualization in this study was plotted using ggplot2 package in R.

### Statistics analysis

All statistics analysis was performed using R software. Students’ t test was used to assess the difference significant between the mean values. All mean values in this study were shown as Mean ± Standard Errors. Outliers were removed by the outliers package in R using the t-Student scores method with 0.95 probability. For the association analysis, two factors whose correlation coefficient > 0.4 or < -0.4 were considered as correlated. And the two factors whose correlation coefficient > 0.2 or < -0.2 were considered as weak correlated. Correlation coefficients were calculated using the Pearson method. The test of the association between the two factors was analyzed using the cor.test function. In order to analyze whether there are differences about the frequencies of CNV/LOH at specific genome positions between adults and aborted fetuses, the method of Fisher exact test in R was used.

## Results

### Summary of the chromosomal microarray data in ART fetuses

**Fig 1:**
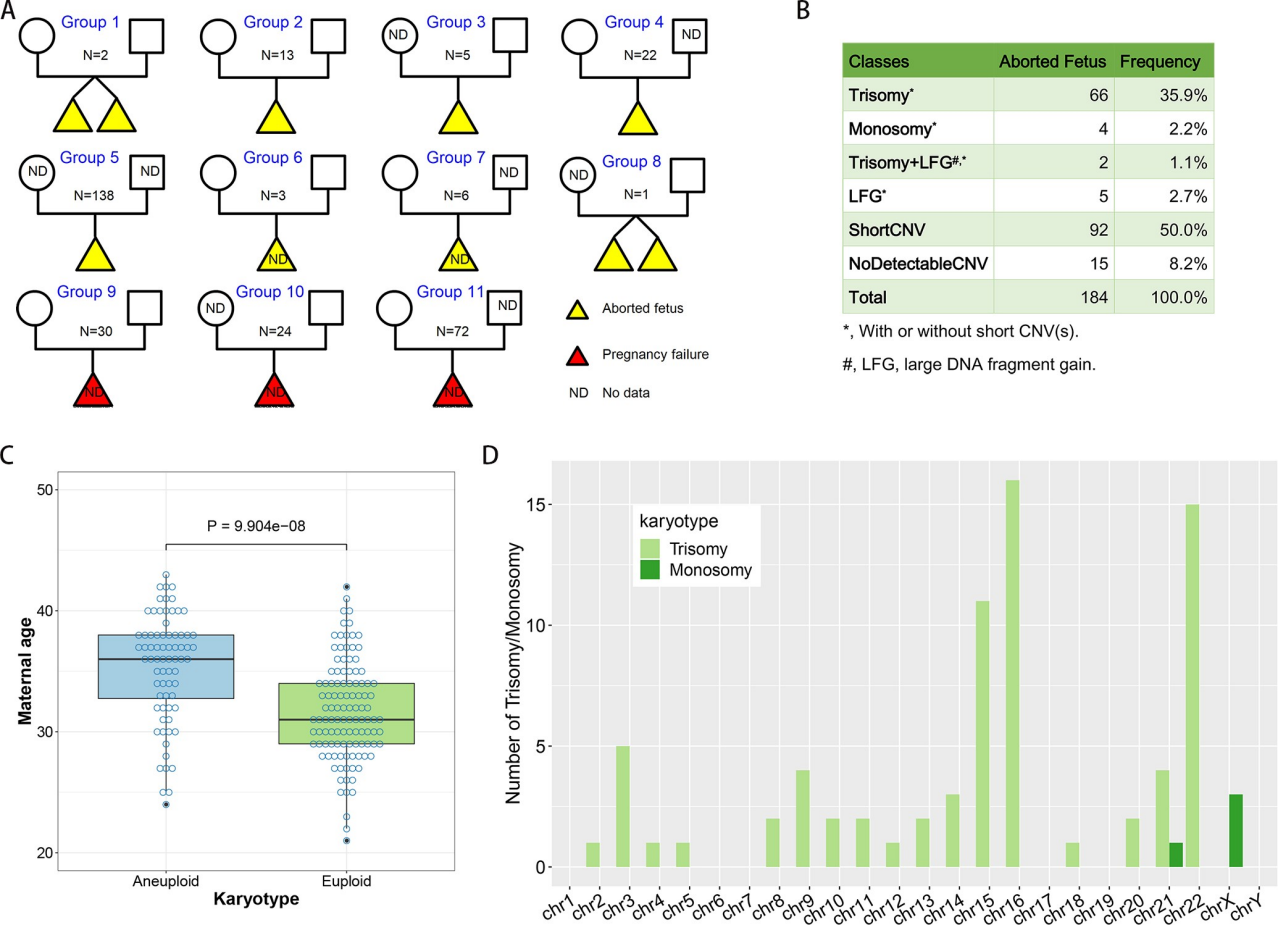
Sample collection and analysis of aneuploidy fetus. (A) Family information of the chromosomal microarray data. (B) Statistics of detected chromatin gains or losses in ART-aborted fetal samples. (C) Aneuploidy of fetus increased with maternal age. (D) The frequencies of chromosome trisomy or monosomy for each chromosome.

To analyze the causes of fetus abortion from the perspective of genomic variations, we analyzed the karyotypes of 184 aborted fetuses during ART, 147 female adult ART patients and 78 male adult patients using the method of CMA. According to the integrality of the pedigree information, we classed the chromosomal microarray data into 11 groups, in which 15 contain full family trio data (group 1 and 2) and others only contain part of family data (Fig 1A and S1 File). From these chromosomal microarray data (S2 File) we found 68 (37.0%) aborted fetuses were trisomy and 4 fetuses (2.2%) are monosomy. There were 5 fetuses (2.7%) having large fragment gains (LFGs, duplication sequence > 4Mbp) and 2 fetuses (1.1%) containing both trisomy and LFGs. For the euploidy aborted fetuses, 92 fetuses (50% of all fetuses) containing short CNVs (length < 4Mbp) and 15 fetuses (8.2%) had no detectable CNVs (Fig 1B).

By analyzing the maternal ages of the ART-aborted fetuses, we found the average maternal ages of aneuploidy aborted fetus (average maternal age 35.36±0.53) was significantly larger than that of euploidy aborted fetus (average maternal age 31.69±0.39, P < 0.01) (Fig 1C). In addition, we analyzed the frequency of 77 numerical aberration events of each chromosome and found chromosome 15 (chr15), chr16 and chr22 were more frequently detected with numerical aberrations. The frequencies of trisomy chr15, chr16 and chr22 were 11 (15.3%), 16 (22.2%) and 15 (20.8%), respectively. The frequency of chromosome losses (1 chr21 loss and 3 chrX losses, 5.2%) was obviously less than that of chromosome gains (94.8%) (Fig 1D).

### Accumulated lengths of CNVs and LOHs are larger in ART-aborted fetuses

**Fig 2:**
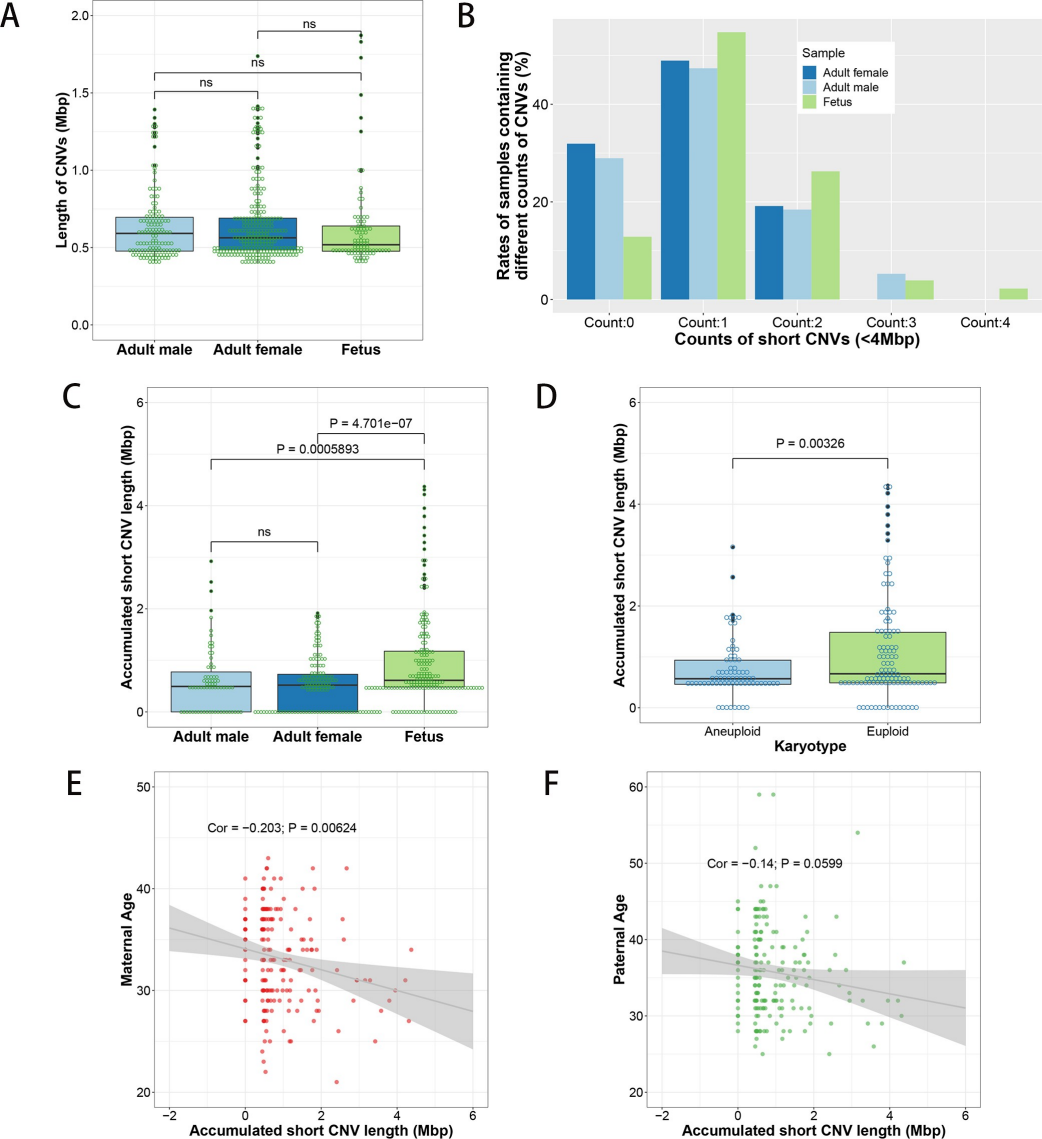
Statistics of short CNVs in ART-aborted fetuses and ART adult patients. (A) Boxplot of lengths of short CNVs of male/female patients and aborted fetuses. (B) Rates of samples containing different number of short CNVs. (C and D) Accumulated lengths of short CNVs in each sample grouped by adult males, adult females and aborted fetuses (C) or by aneuploid and euploid of the aborted fetuses (D). (E and F) The association analysis of accumulated short CNV lengths with maternal ages (E) and paternal ages (F). ‘ns’, P values of t test > 0.05. ‘Cor’, correlation coefficient.

In addition to numerical aberrations of chromosomes, CNVs and LOHs were also detected by the CMA. As ultra-large CNVs might induce fetus death with the same mechanism as chromosome gains or losses, here we just analyzed the short CNVs whose lengths were less than 4 Mbp. From the chromosomal microarray data we found that there was no significant difference about the lengths of these short CNVs between adult male/female ART patients and the ART-aborted fetuses (mean lengths of CNVs for male patients, female patients and aborted fetuses were 0.63±0.03Mbp, 0.64±0.02Mbp and 0.64±0.02Mbp respectively) (Fig 2A). However, the average number of CNVs in fetuses (1.28±0.06 CNVs per fetus) was larger than that in adult males (1.00±0.10, p < 0.01) and adult females (0.87±0.06, p < 0.01) (S3 File and Fig 2B), and the average of the accumulated CNV lengths in ART-aborted fetuses (0.97±0.07Mbp) was significantly longer than that of adult males (0.61±0.08Mbp, p < 0.01) and adult females (0.55±0.04Mbp, p < 0.01) (Fig 2C). In addition, we found the euploidy aborted fetuses contained larger accumulated CNV lengths (1.11±0.10Mbp) than that of aneuploidy aborted fetuses (0.75±0.07Mbp, p < 0.01) (Fig 2D). To analyze the correlation between accumulated CNV lengths and parental ages, we calculated the correlation coefficients of accumulated CNV lengths and parental ages. As a result, the correlation coefficient of accumulated CNV lengths with maternal ages was -0.203 (P < 0.01), and that of accumulated CNV lengths with paternal ages was -0.14 (P > 0.05), indicating that there is a weak negative correlation between maternal ages and the accumulated CNV lengths in ART-aborted fetuses, but there is no significant association between accumulated CNV lengths in ART-aborted fetuses with paternal ages (Fig 2E and 2F).

**Fig 3:**
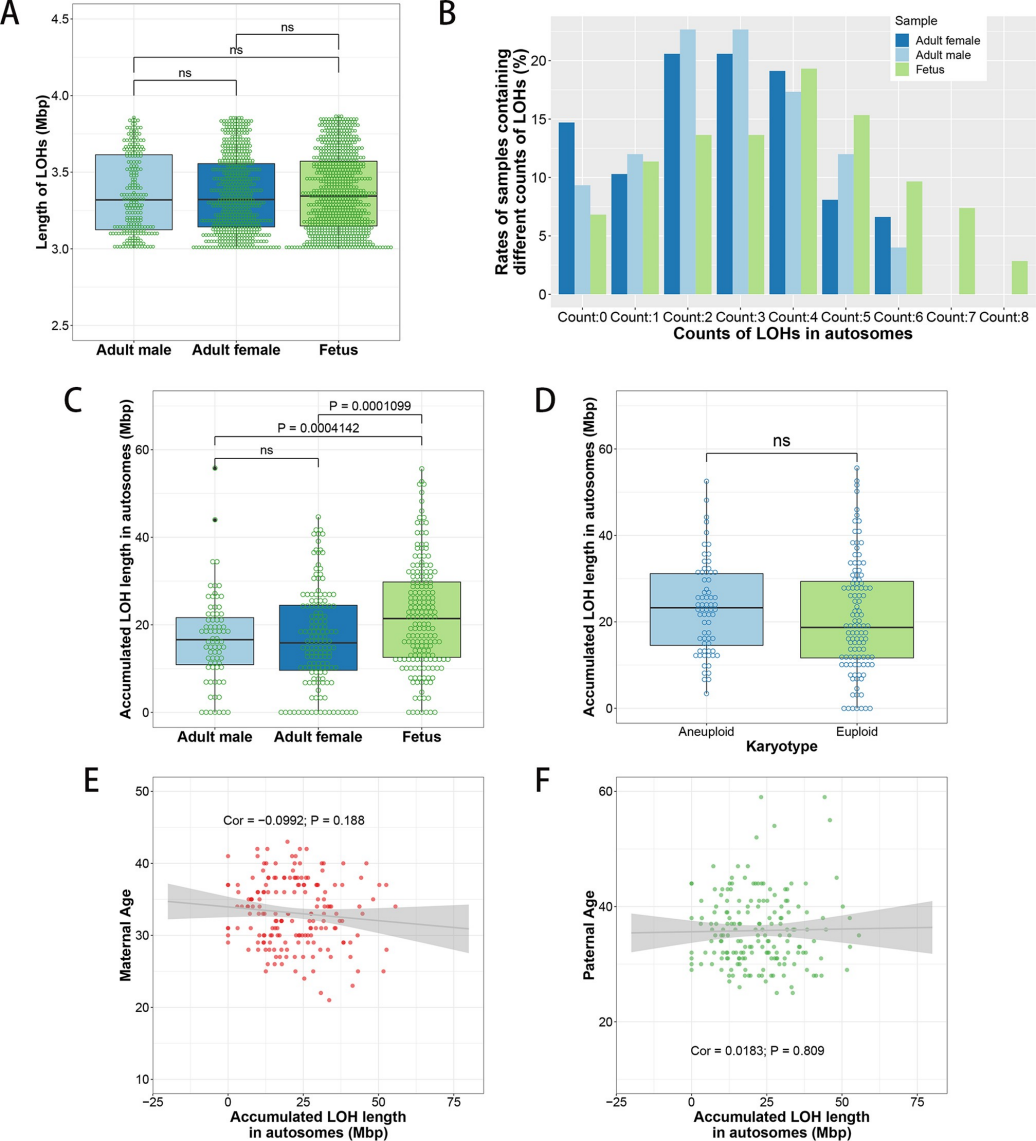
Statistics of autosomal LOHs in ART-aborted fetuses and ART adults. (A) Boxplot of lengths of autosomal LOHs of male/female patients and the ART-aborted fetuses. (B) Rates of samples containing different number of autosomal LOHs. (C and D) Accumulated lengths of autosomal LOHs in each sample grouped by adult males, adult females and aborted fetuses (C) or by aneuploid and euploid of the aborted fetuses (D). (E and F) The association analysis of accumulated autosomal LOH lengths with maternal ages (E) and paternal ages (F). ‘ns’, P values of t test > 0.05. ‘Cor’, correlation coefficient.

Similarly, we also analyzed the average lengths of autosomal LOHs in male adults (3.36±0.02 Mbp), female adults (3.36±0.01 Mbp) and aborted fetuses (3.37±0.01 Mbp). We found that there is no significant difference about the mean LOH lengths among the three groups (Fig 3A). However, the average number of autosomal LOHs in each fetus was 3.66±0.16, which was significantly higher than that in male adults (2.79±0.18, p < 0.01) and female adults (2.70±0.15, p < 0.01) (S3 File and Fig 3B). The average accumulated length of LOHs in ART-aborted fetuses was 21.90±0.91 Mbp, which was significantly longer than the average accumulated length of LOHs in male adults (17.33±1.40, p < 0.01) and female adults (17.70±1.28, p < 0.01) (Fig 3C). We hadn’t found difference about accumulated LOH lengths between aneuploidy ART-aborted fetuses (23.27±1.27 Mbp) and euploidy ART-aborted fetuses (21.03±1.25 Mbp) (Fig 3D). The correlation coefficients of accumulated LOH lengths in ART-aborted fetuses with maternal ages was -0.099 (Fig 3E) and that of ART-aborted fetal accumulated LOH lengths with paternal ages was 0.018 (Fig 3F), indicating that no association exists between the ART-aborted fetal accumulated LOH lengths and the parental ages.

### Chromosomal positions with higher frequency of CNV or LOH in the ART-aborted fetuses

**Fig 4:**
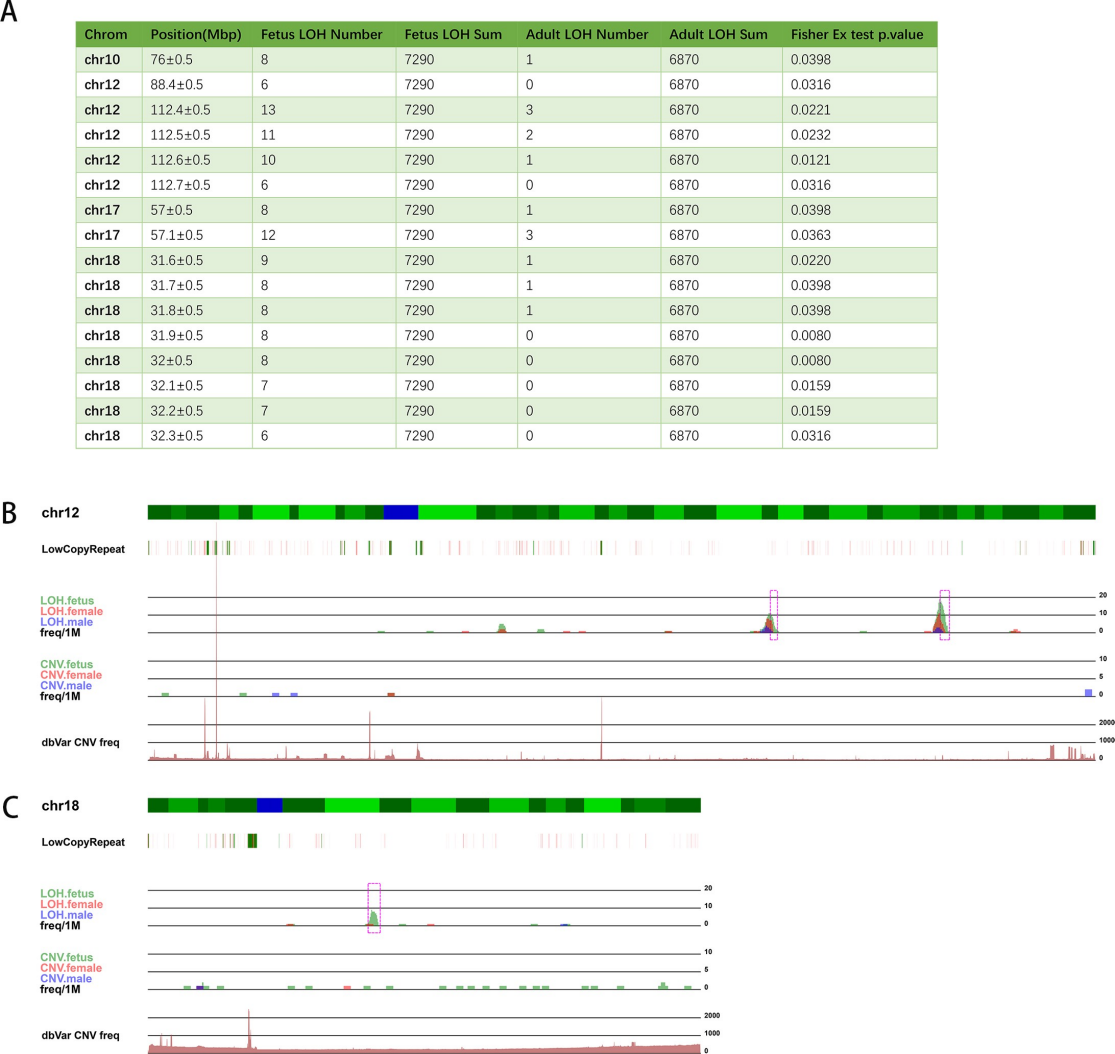
LOHs showed higher occur frequency in ART-aborted fetuses. (A) Chromosomal positions where high frequency of LOH occurred in aborted fetuses. (B) Visualization of the positions in chr12 and chr18 where LOHs were highly occurred in aborted fetuses.

To analyze whether there was a specific chromosomal position where CNV or LOH frequency is higher in ART-aborted fetus than adults, we plotted the CNV and LOH frequency data in this study and CNV data from dbVar database (S4 File). As a result, we hadn’t found a specific CNV in ART-aborted fetus showed higher frequency of occurrence than adults, but there were some chromosomal regions which showed higher LOH occur frequencies in aborted fetus than that in the adults (Fisher exact test p value < 0.05, Fig 4A). From the visualized LOH peaks (Fig 4B and 4C) we could see that the scale of LOH peaks were larger in aborted fetus compared with that in adults.

## Discussion

The individual CNVs could be inherited from parents or be generated *de novo* during gametogenesis and/or embryogenesis. Large size or number of CNVs had been shown to be associated with developmental delay [24] and/or mental retardation [25]. In this study we found that the genomes of aborted fetuses during ART contains more short CNVs (< 4 Mbp) compared with the adult genomes. As CNVs were generally generated by DSBs or replication stresses [12, 15], the increased number of CNVs in aborted ART-fetuses might indicate that during the gametogenesis or embryogenesis, a higher dose of DNA DSBs had attacked these fetuses. Unlike CNVs, LOHs could be generated by consanguineous marriage or by break-induced DNA replication of non-sister homologous chromatid [18] during DNA DSB repair. The break-induced replication events of non-sister homologous chromatids couldn’t occur in gametes, so LOHs detected in the fetuses of non-consanguineous marriage parents might be formed after fertilization. If the LOH formed in embryonic cells after several mitotic cell cycles, the detection of LOHs would become difficult to be detected as the converted SNPs would only occur in a small proportion of cells. So the increased LOH number in ART-aborted fetuses indicates that the ART-aborted fetuses might have experienced more DNA DSBs during early embryo development than normal fetuses. Speculatively, during the gametogenesis or embryogenesis progression of the ART-aborted fetuses, their genomes might have experienced more DNA DSBs or other damages than the normal fetuses, and the accumulated CNVs and LOHs might be the causing factor for the final fetal abortion.

In this study, we found there is a weak negative correlation between maternal ages and the accumulated CNV lengths in ART-aborted fetuses. On the one hand, this weak correlation might be generated by the lack of samples. On the other hand, the accumulated CNV lengths might be more detrimental to the embryos/fetuses in aged mothers and induce abortion. Correspondingly, we found the accumulated CNV lengths were longer in euploid aborted fetuses than that in aneuploid aborted fetuses. As fetal aneuploid is mainly increased with maternal age, the decrease of CNV accumulated lengths in aneuploid aborted fetuses might corroborate that there is negative correlation between maternal ages and accumulated CNV lengths in aborted fetuses. In our study, we found there is no significant correlation of paternal ages with accumulated CNV lengths in ART-aborted fetuses. And there is also no significant correlation between parental ages and accumulated LOH lengths in ART-aborted fetuses. These results indicated that the accumulated CNV and LOH lengths were not associated with paternal ages and parental ages respectively.

Considering the potential pathogenicity of CNVs and LOHs, the abortion of ART-fetuses might be caused by the accumulation of adverse effects of the increased CNVs or LOHs. On the other hand, the accumulation of CNVs/LOHs in ART-aborted fetuses might be just a parallel result but not a direct reason of the abortion. Evidences had shown that DNA damage in either oocytes [26–29] or sperms [30–33] could induce spontaneous abortion. So DNA damage can be the source of both CNVs/LOHs and abortion. Then the excessive CNVs and LOHs in ART-aborted fetuses may be parallelly produced by the excessive DNA damage occurred during gametogenesis or early embryo development. Speculatively, the DNA DSBs in gametes or early embryos might affect the fetal developmental competency by generating pathogenic mutations, altering the epigenetic modifications [34] or delaying the cell division [35]. Compared to normal embryos, somatic cell nuclear transfer (SCNT) created embryos has a higher rate of fetal death. Evidences had showed that decreasing the H3K9me3 level in early SCNT embryos would not only increase the blastocyst rates but also increase the pup rates, which indicate that high level of H3K9me3 might block the fetal development whereas low level of H3K9me3 is beneficial for fetal development [36]. H3K9me3 is a heterochromatin marker which could be induced by DSBs [37] and cause transcription repression in chromatin [38]. So high level of DNA DSBs in early embryos might increase the H3K9me3 modification and thus impair the subsequent fetal development.

In conclusion, in this study we found that there was an increase of number and accumulated lengths of CNVs and LOHs in ART-aborted fetuses. So to decrease the abortion rate during ART, it might be extremely important to investigate the source of embryonic genome instability and prevent the DNA damage of fetal genomes.

## Supporting information

S1 File Sample information of the chromosomal microarray data. (XLSX)

S2 File Positions of chromosomal variants in the chromosomal microarray data. (XLSX)

S3 File CNV and LOH numbers of each sample. (XLSX)

S4 File CNV and LOH peaks in human genome. (PDF)
