# Correction: Chromosomal variants accumulate in genomes of the spontaneous aborted fetuses revealed by chromosomal microarray analysis

**DOI:** 10.1371/journal.pone.0263099

**Published:** 2022-01-21

**Authors:** Sen Li, Lei-Ning Chen, Xing-Hua Wang, Hai-Jing Zhu, Xiao-Long Li, Xie Feng, Lei Guo, Xiang-Hong Ou, Jun-Yu Ma

The second author’s name is spelled incorrectly. The correct name is: Lei-Ning Chen. The correct citation is: Li S, Chen L-N, Wang X-H, Zhu H-J, Li X-L, Feng X, et al. (2021) Chromosomal variants accumulate in genomes of the spontaneous aborted fetuses revealed by chromosomal microarray analysis. PLoS ONE 16(11): e0259518. https://doi.org/10.1371/journal.pone.0259518
