# Supplementary figures and images for: Chromosomal variants accumulate in genomes of the spontaneous aborted fetuses revealed by chromosomal microarray analysis
Source: PLoS One. 2021 Nov 2;16(11):e0259518. doi: 10.1371/journal.pone.0259518 (PMC8562782; doi:10.1371/journal.pone.0259518)

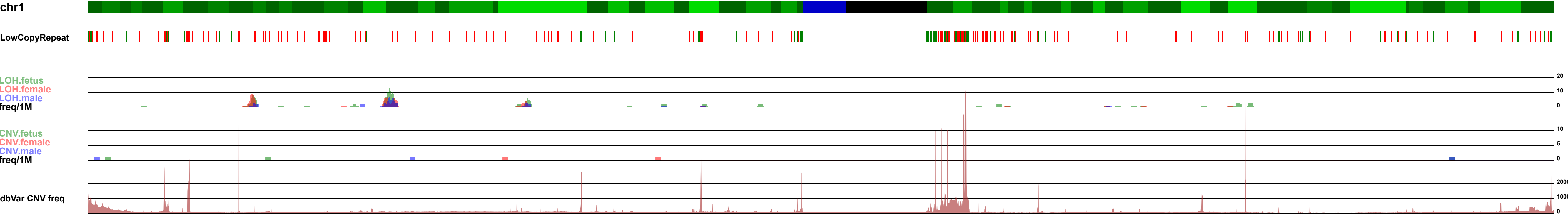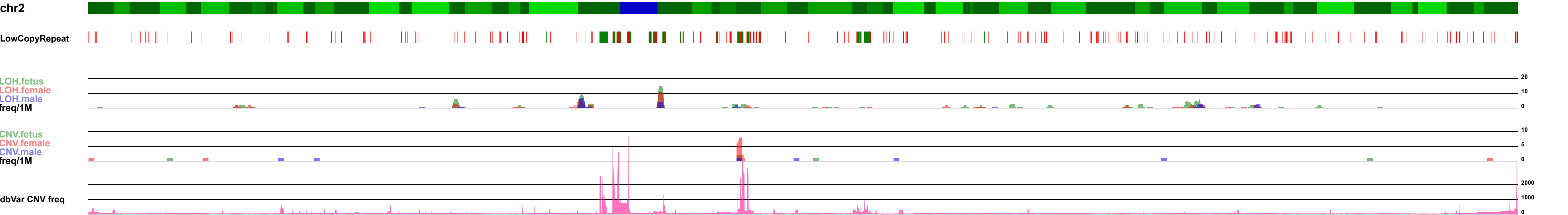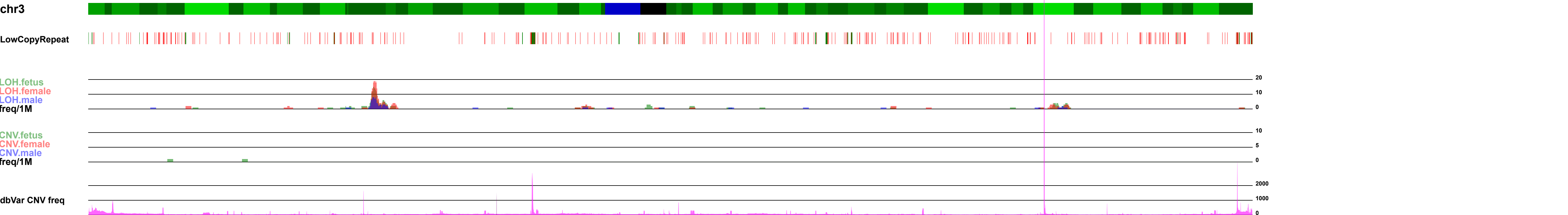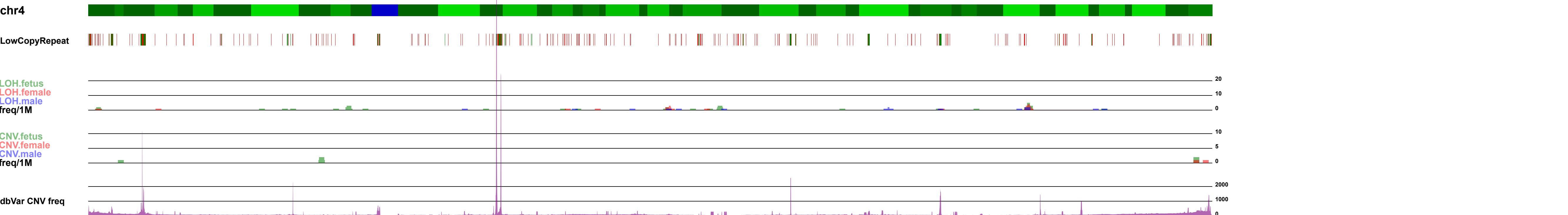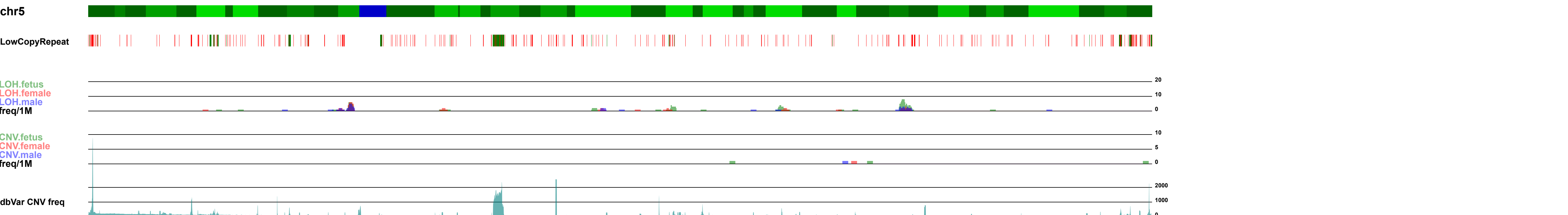

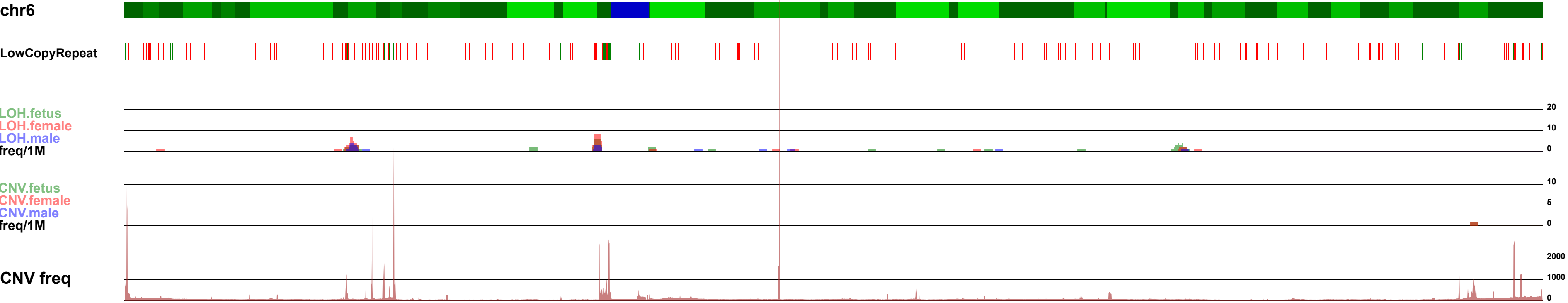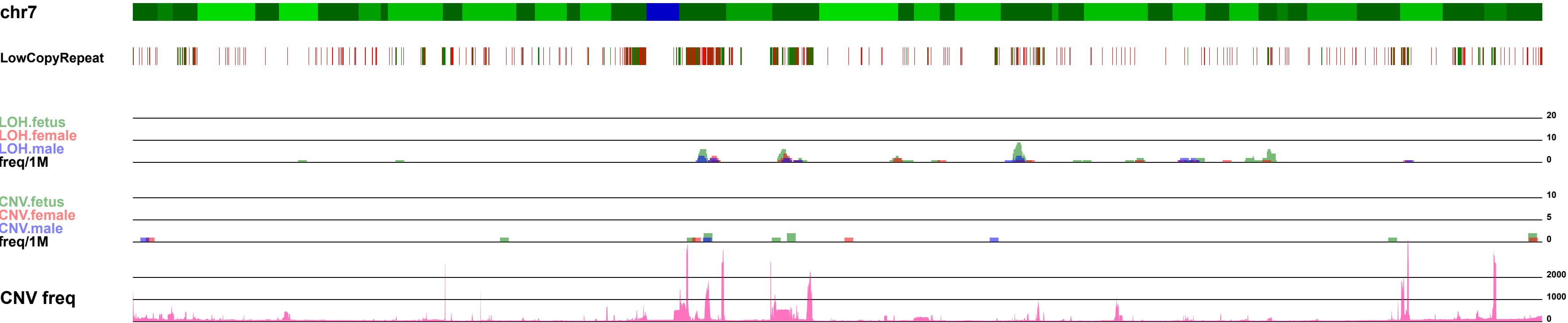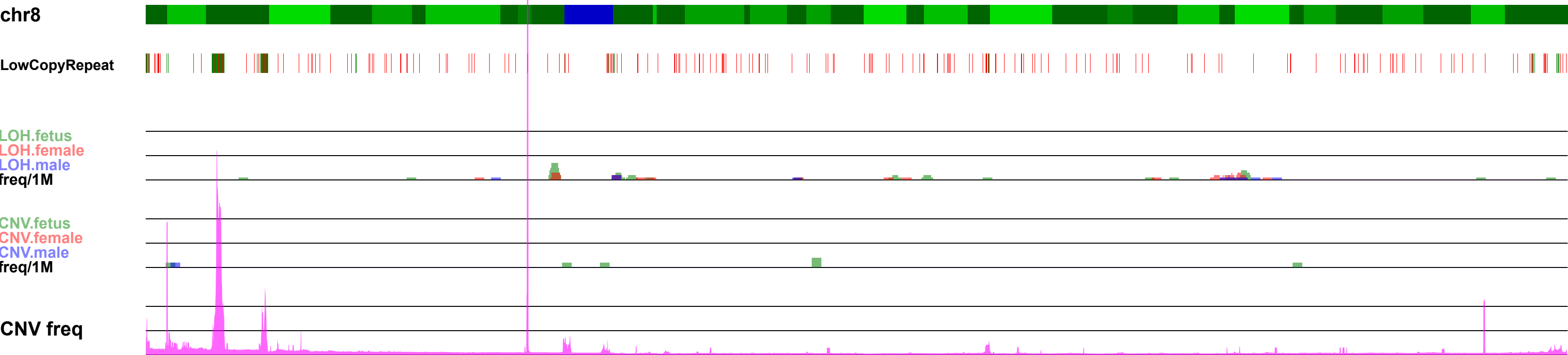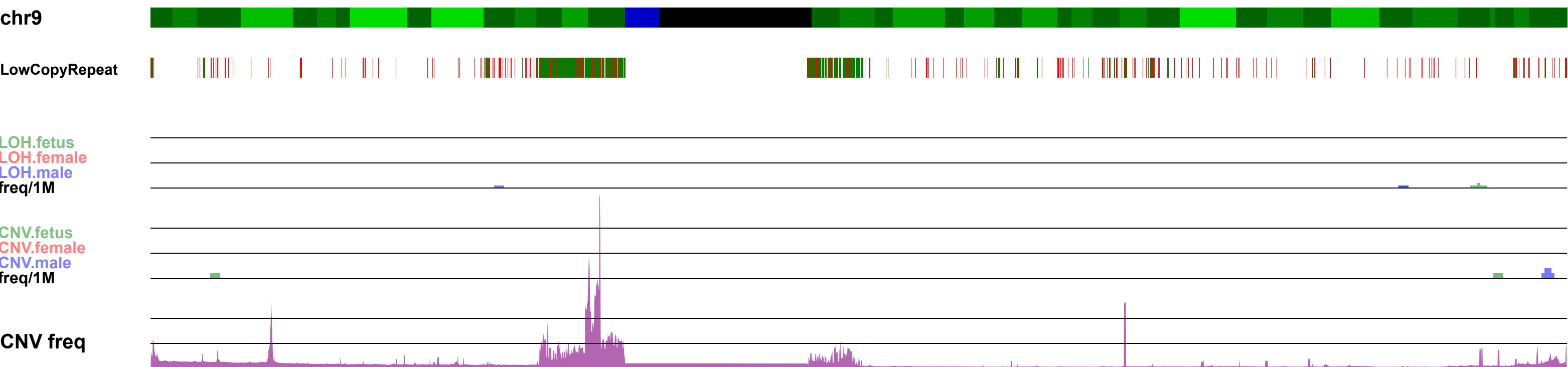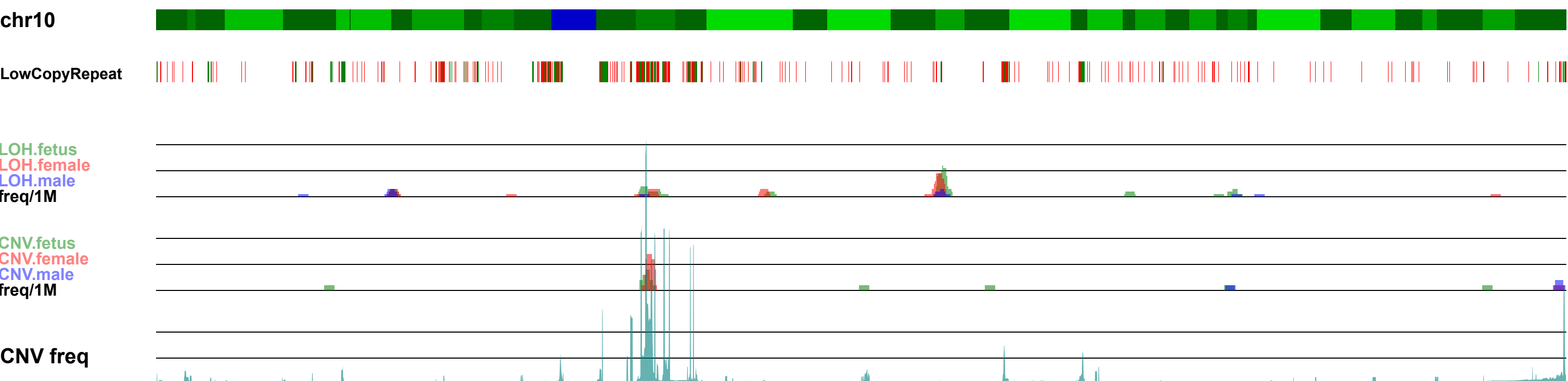

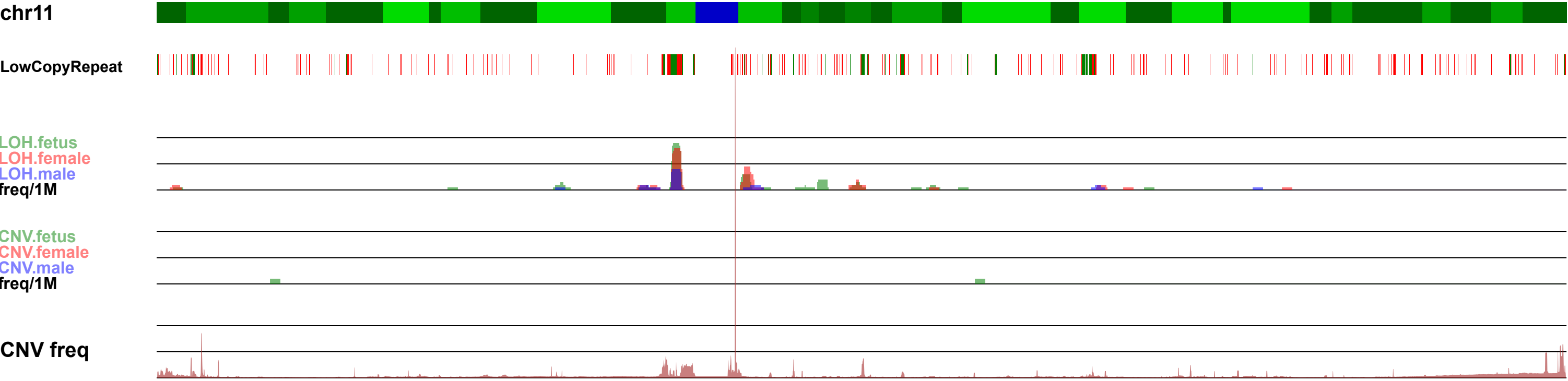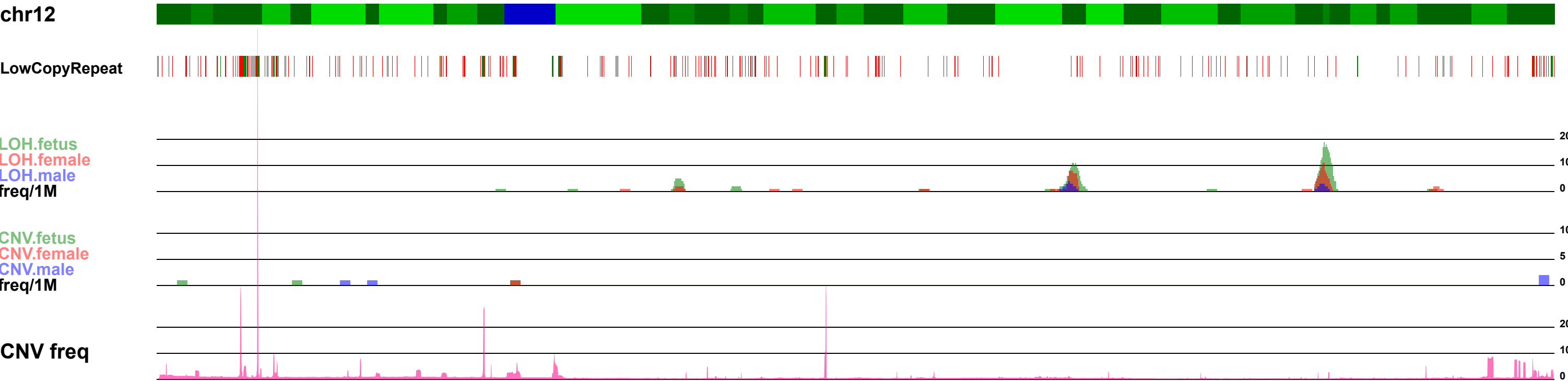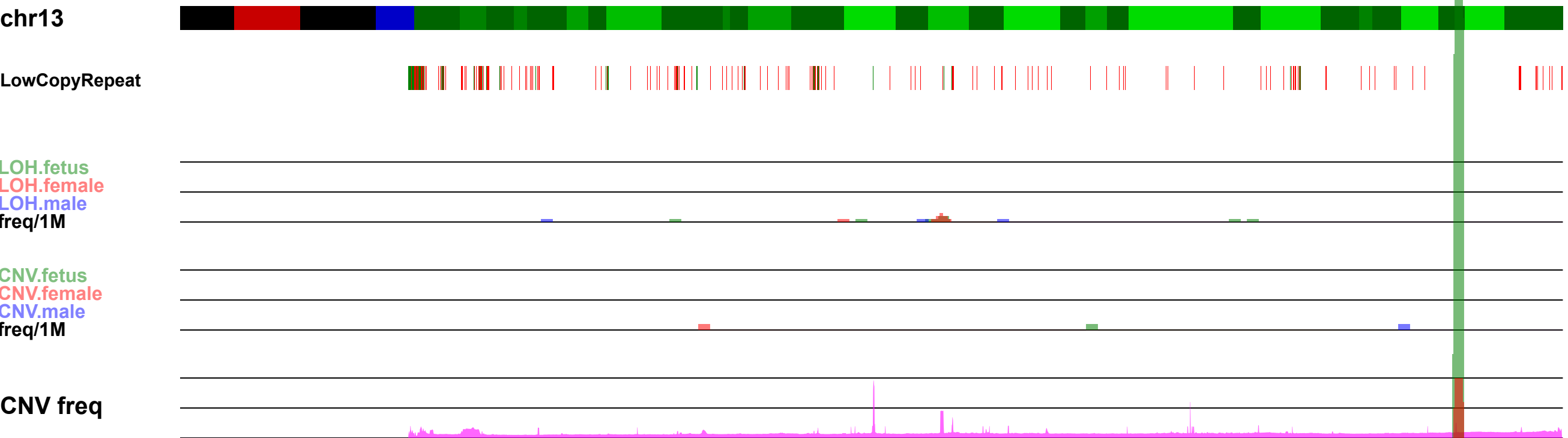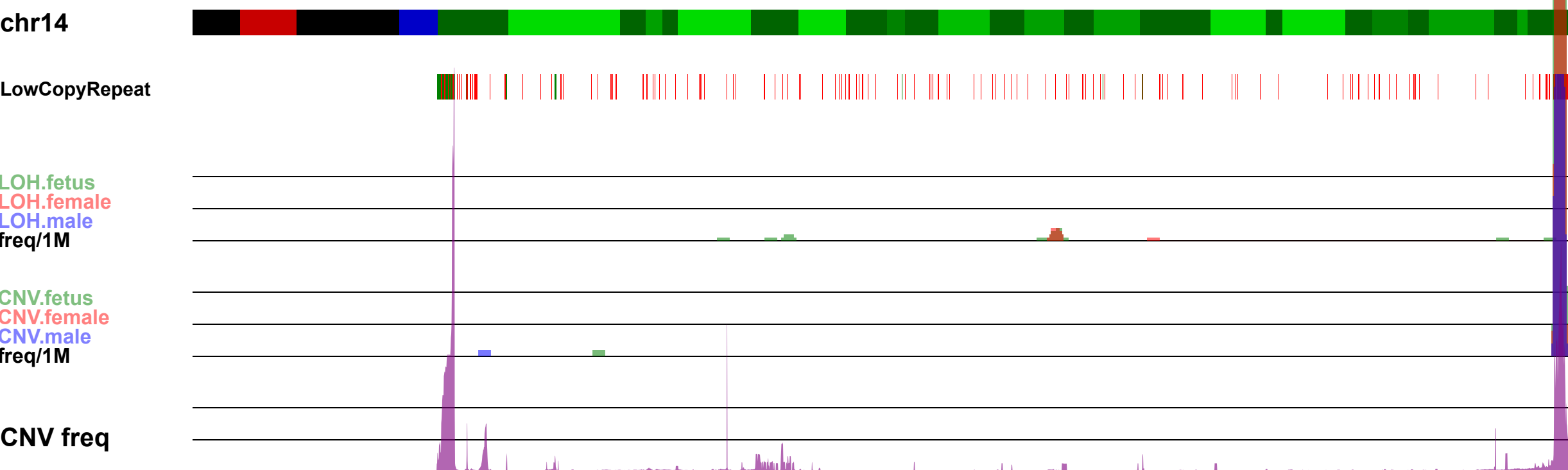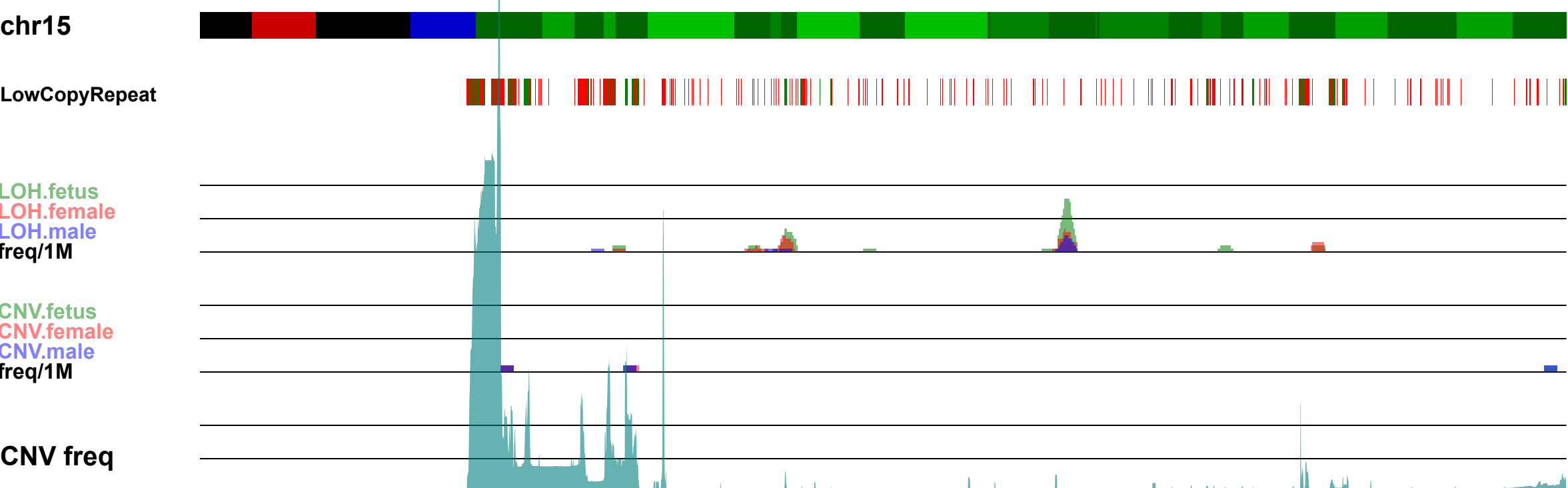

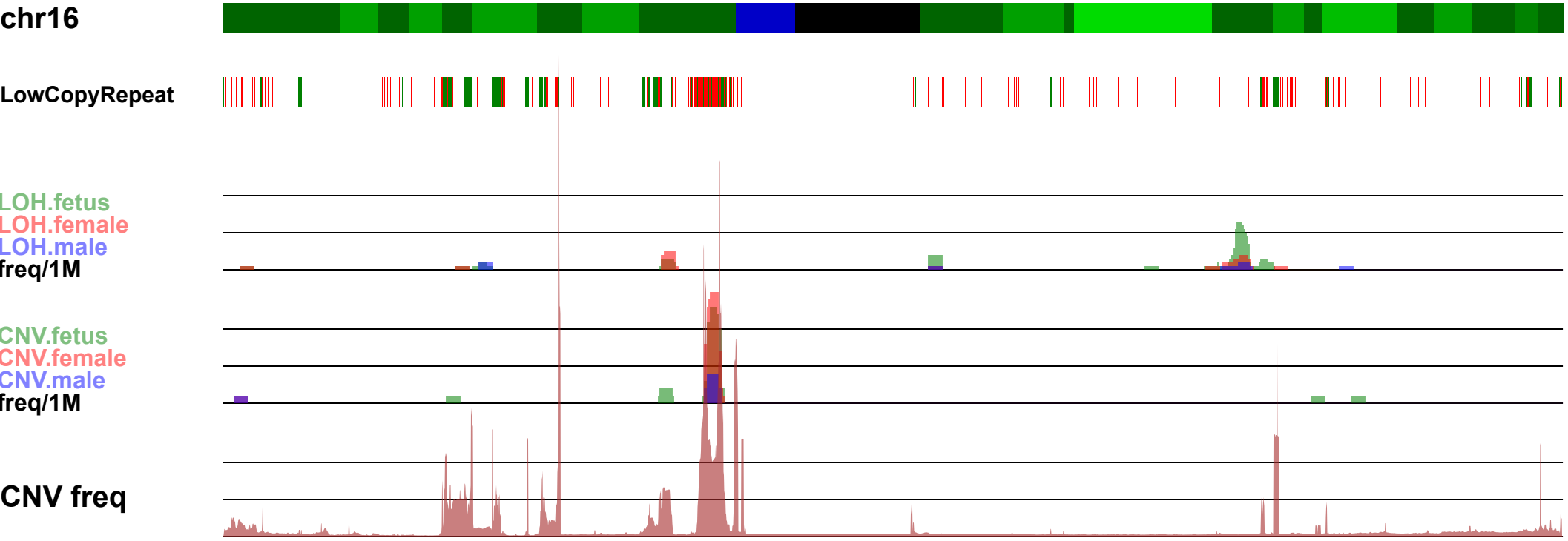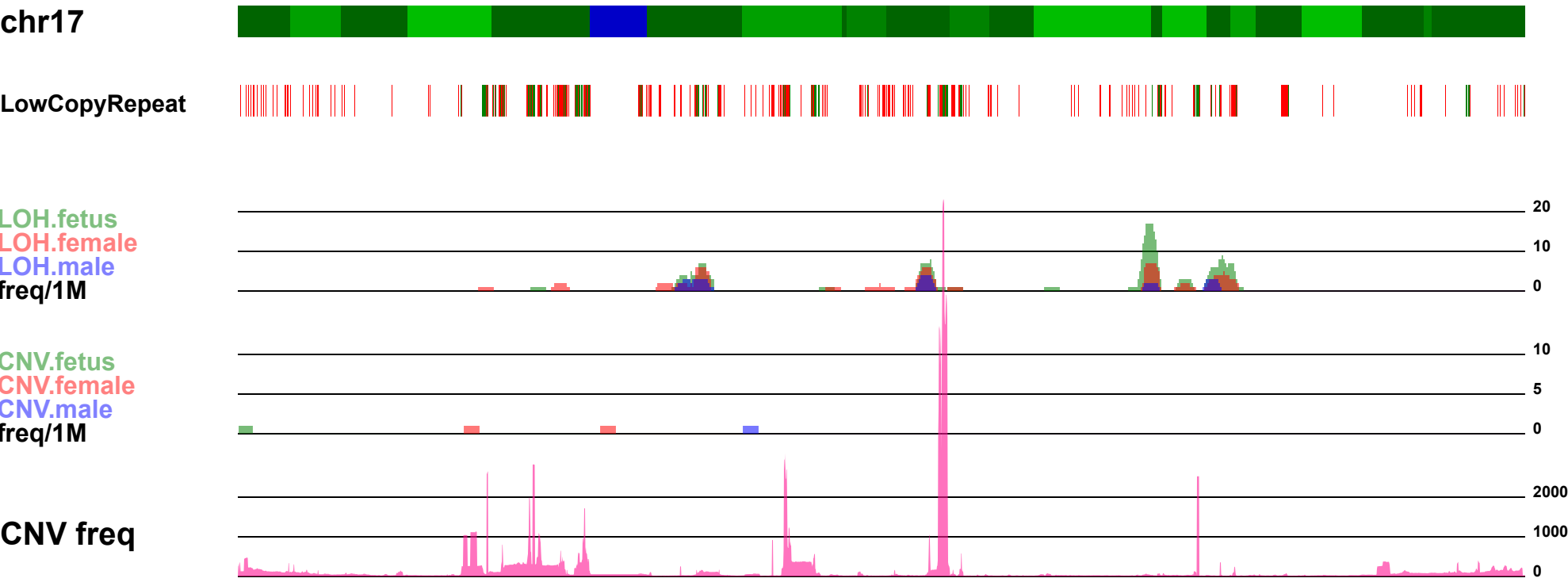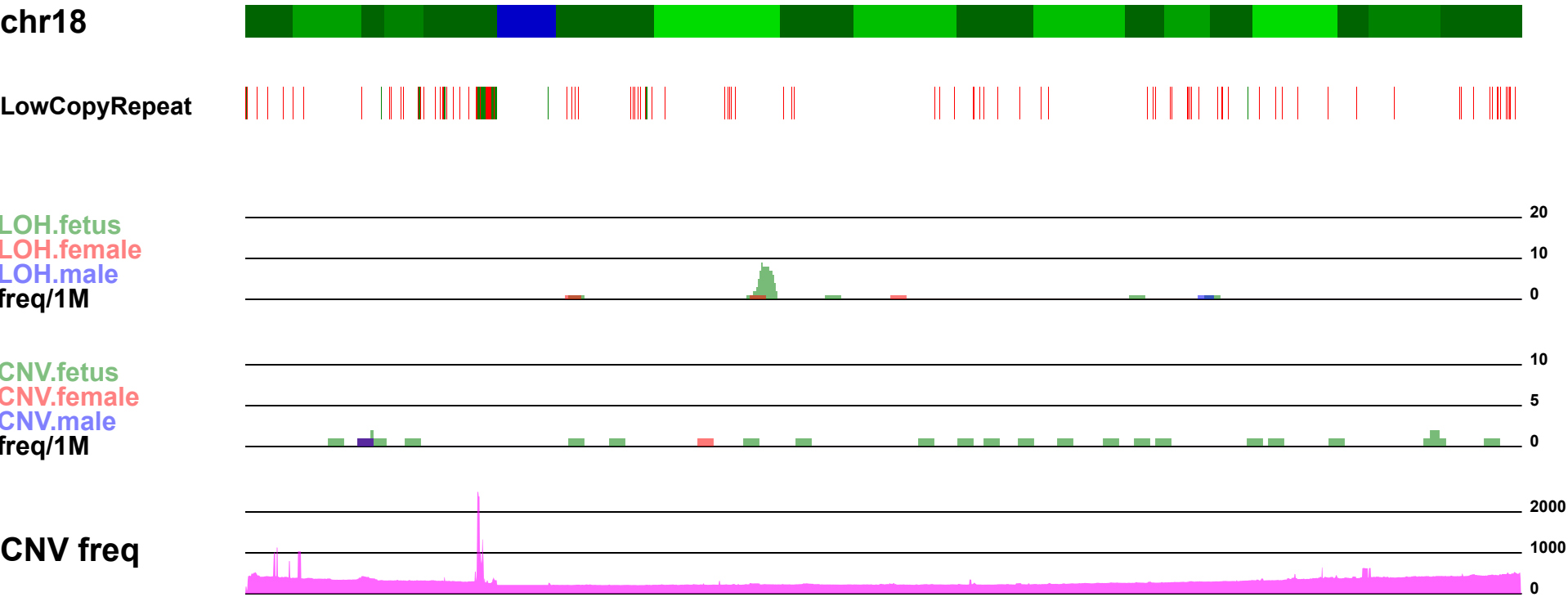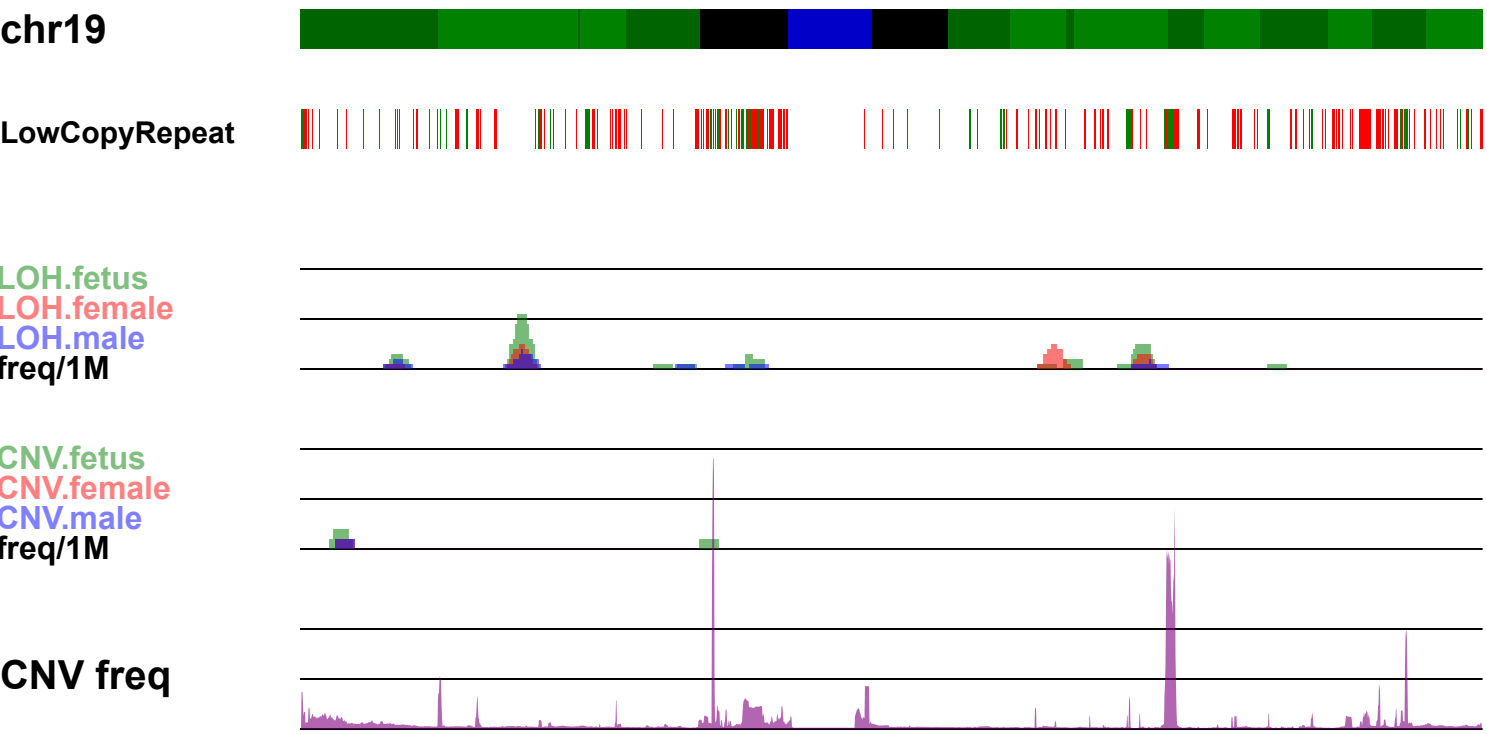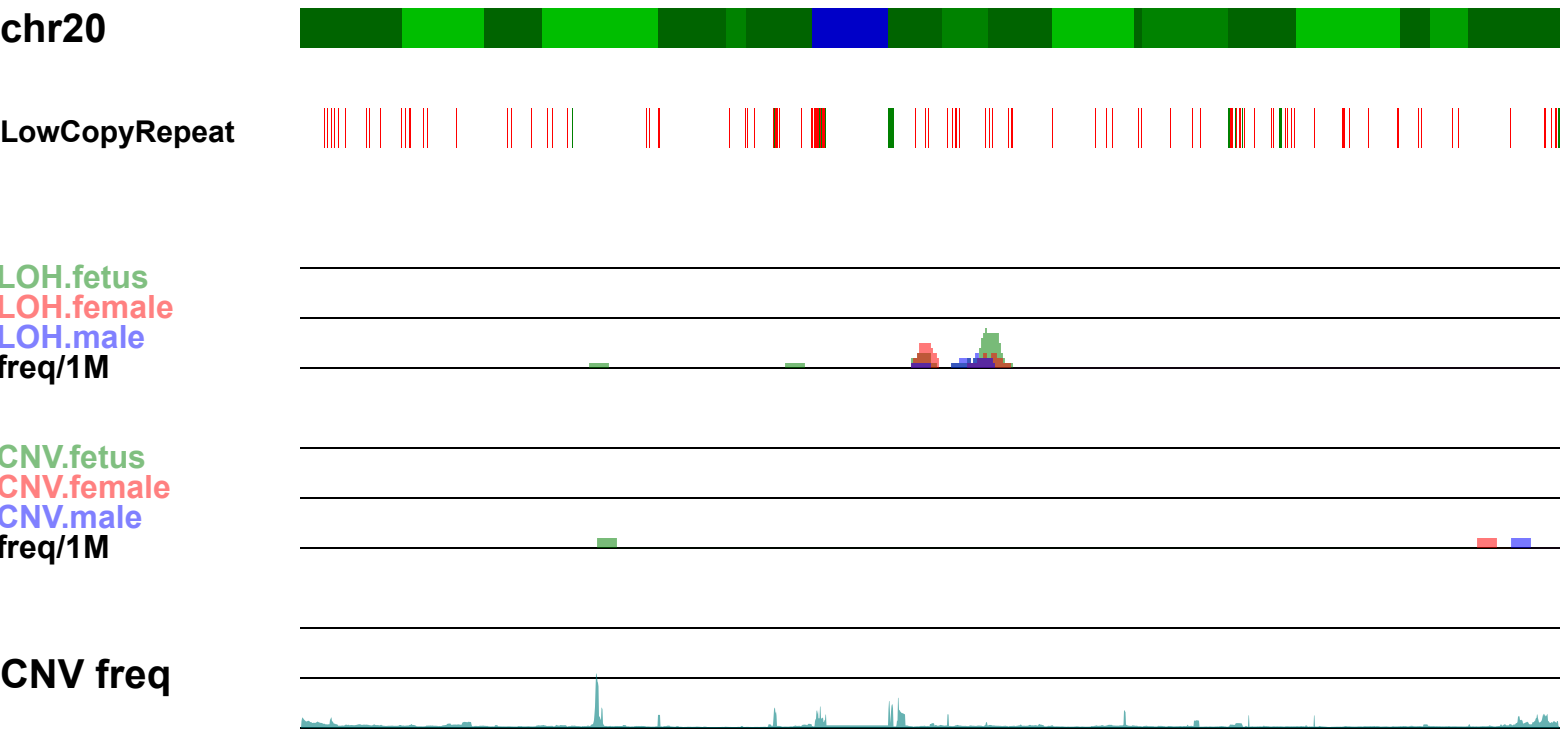

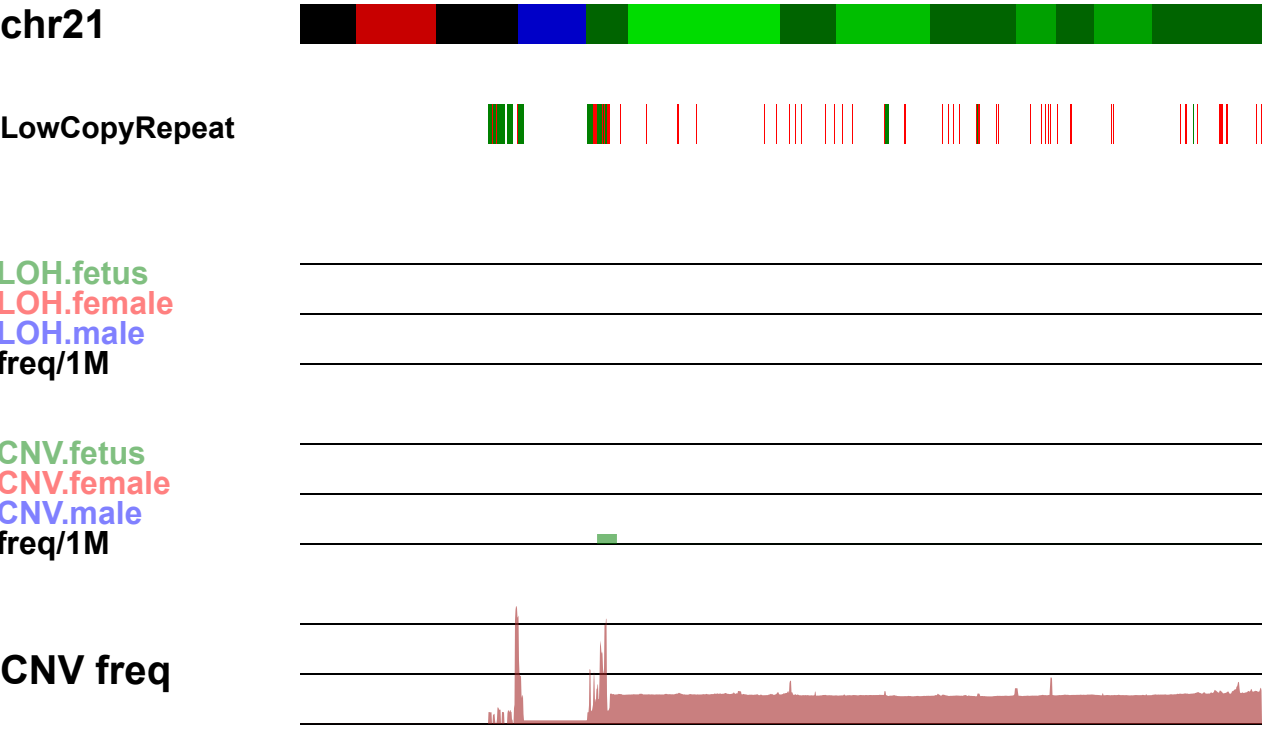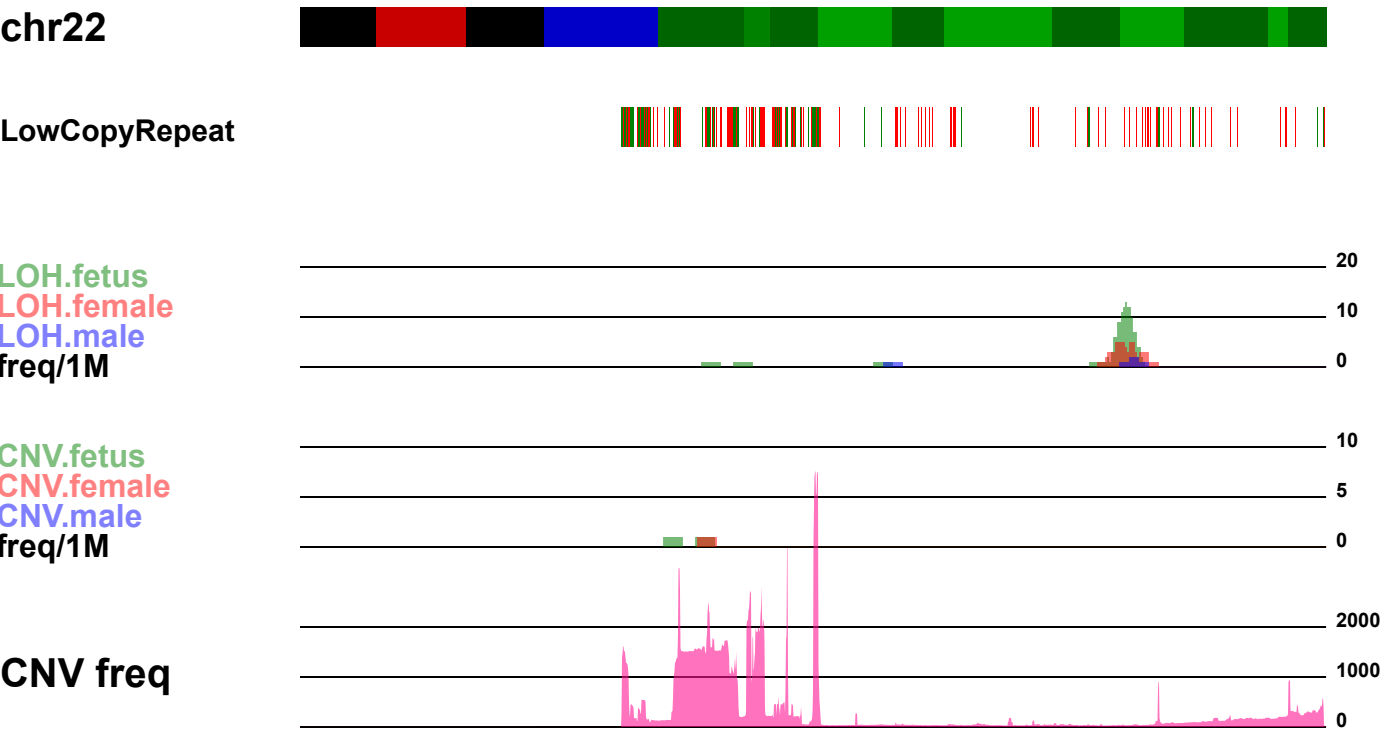

Supplement: S4 File — (PDF) [file pone.0259518.s004.pdf]
